# Functional Roles of p38 Mitogen-Activated Protein Kinase in Macrophage-Mediated Inflammatory Responses

**DOI:** 10.1155/2014/352371

**Published:** 2014-03-20

**Authors:** Yanyan Yang, Seung Cheol Kim, Tao Yu, Young-Su Yi, Man Hee Rhee, Gi-Ho Sung, Byong Chul Yoo, Jae Youl Cho

**Affiliations:** ^1^Department of Genetic Engineering, Sungkyunkwan University, Suwon 440-746, Republic of Korea; ^2^Division of Gynecologic Oncology Department of Obstetrics and Gynecology, Ewha Womans University Mokdong Hospital College of Medicine, Ewha Womans University, Seoul 158-710, Republic of Korea; ^3^College of Veterinary Medicine, Kyungpook National University, Daegu 702-701, Republic of Korea; ^4^Department of Herbal Crop Research, National Institutes of Horticultural & Herbal Science, Rural Development Administration, Suwon 441-707, Republic of Korea; ^5^Colorectal Cancer Branch, Research Institute, National Cancer Center, Goyang, Gyeonggi 410-769, Republic of Korea

## Abstract

Inflammation is a natural host defensive process that is largely regulated by macrophages during the innate immune response. Mitogen-activated protein kinases (MAPKs) are proline-directed serine and threonine protein kinases that regulate many physiological and pathophysiological cell responses. p38 MAPKs are key MAPKs involved in the production of inflammatory mediators, including tumor necrosis factor-**α** (TNF-**α**) and cyclooxygenase-2 (COX-2). p38 MAPK signaling plays an essential role in regulating cellular processes, especially inflammation. In this paper, we summarize the characteristics of p38 signaling in macrophage-mediated inflammation. In addition, we discuss the potential of using inhibitors targeting p38 expression in macrophages to treat inflammatory diseases.

## 1. Introduction

Inflammatory response is a basic protective immune process of the organism and is accompanied by symptoms such as redness, heat, swelling, and pain associated with damage to tissues or organs [1]. This is one of the mechanisms by which our body defends us from pathogens such as parasites, bacteria, viruses, and other harmful microorganisms. Diseases induced by chronic inflammation, including gastritis, colitis, dermatitis, rheumatoid arthritis, pulmonary diseases, and type II diabetes, damage millions of people's health every year. Of concern is the increase in prevalence of these chronic inflammatory diseases. Furthermore, there is growing evidence that inflammation is a critical initiation factor inducing a variety of other major diseases such as cancer, atherosclerosis, Alzheimer's disease, cardiovascular disease, neurological disorders, and pulmonary diseases [2–7]. Therefore, a better understanding of inflammation is clinically significant and could improve treatment strategies.

Macrophages within tissues play an essential role in the initiation, development, and resolution of inflammation [8–11]. Macrophages are white blood cells that are differentiated from monocytes. Their roles are to clean up damaged cells and pathogens by phagocytosis and to activate immune cells, such as neutrophils, dendritic cells, macrophages, and monocytes, in response to pathogens and diseases. They can be activated or deactivated during inflammatory processes depending on the signaling molecules produced. Stimulation signals include lipopolysaccharide (LPS), cytokines (interleukin-1 (IL-1) and tumor necrosis factor-*α* (TNF-*α*)), other chemical mediators, and extracellular matrix proteins. A variety of membrane receptors are expressed on the surfaces of macrophages, including pattern recognition receptors (PRRs) such as dectin-1 and Toll-like receptors (TLRs) [12, 13]. These receptors recognize activation signals and subsequently activate downstream protein kinases, eventually resulting in the stimulation of transcription factors including activator protein-1 (AP-1), nuclear factor-kappa B (NF-*κ*B), and cAMP response element-binding protein (CREB).

Various intracellular proteins can initiate inflammation. p38 proteins are a class of mitogen-activated protein kinases (MAPKs) that are major players during inflammatory responses, especially in macrophages. p38, also called RK or cytokinin-specific binding protein (CSBP), was identified in 1994 and is the mammalian ortholog of the yeast Hog1p MAP kinase [14]. p38 was isolated as a 38 kDa protein that is rapidly phosphorylated at a tyrosine residue in response to LPS stimulation, and the p38 gene was cloned through binding of the p38 protein with pyridinyl imidazole derivatives [15]. p38 expression is upregulated in response to inflammatory and stress stimuli, such as cytokines, ultraviolet irradiation, osmotic shock, and heat shock, and is involved in autophagy, apoptosis, and cell differentiation [16–20]. Accumulating evidence suggests that p38 plays an important role in arthritis and inflammation of the liver, kidney, brain, and lung and that it acts as a critical player in inflammatory diseases mediated by macrophages [21–23].

In this paper, we summarize the characteristics of p38 and highlight the physiological significance of p38 activation in macrophage-mediated inflammatory responses. Moreover, we discuss the possibility of using plant extracts, natural products, and chemicals that target p38 as therapeutic drug candidates for the treatment of inflammatory diseases.

## 2. Structure and Function of p38 Kinases

### 2.1. The p38 Family

p38 family members are classified into four subtypes: *α* (MAPK14), *β* (MAPK11), *γ* (MAPK12/ERK6), and *δ* (MAPK13/SAPK4) (Table 1). Genes encoding p38*α* and p38*β* show 74% sequence homology, whereas *γ* and *δ* are more distant relatives, with approximately 62% sequence identity [24–26]. Genes encoding p38*α* and p38*β* are ubiquitously expressed within tissues, and especially highly expressed in heart and brain. However, p38*γ* and p38*δ* show tissue-specific expression patterns; p38*γ* is highly expressed in skeletal muscle, whereas p38*δ* expression is concentrated in the kidneys, lungs, pancreas, testis, and small intestine [27]. In addition, p38*γ* expression can be induced during muscle differentiation, and its expression can also be developmentally regulated. Moreover, we demonstrated very high expression of the active form of p38 in inflammatory diseases, such as gastritis, colitis, arthritis, and hepatitis [28, 29] (unpublished data). p38*α* and p38*δ* are abundantly expressed in macrophages, whereas p38*β* is undetectable. p38*α* and p38*δ* are also expressed in endothelial cells, neutrophils, and CD4+ T cells, whereas p38*β* is abundant in endothelial cells. These findings indicate that, even though the four p38 family members share sequence homology, their expression is cell- and tissue dependent and their functions may therefore be different.

### 2.2. p38 Structure and Domains

p38 kinases have two domains: a 135 amino acid N-terminal domain and a 225 amino acid C-terminal domain. The main secondary structure of the N-terminal domain is *β*-sheets, while the C-terminal domain has a *α*-helical structure. The catalytic site is located in the region linking the two domains. The phosphorylation lip of p38 consists of 13 residues, Leu-171–Val-183, and the protein is activated by phosphorylation of a single threonine (Thr-180) and a single tyrosine residue (Tyr-182) in the lip [30]. Moreover, in* Drosophila* p38 MAPK, phosphorylation of tyrosine-186 was detected exclusively in the nucleus following osmotic stress [31]. p38 isoforms show various three-dimensional structures with differences in the orientation of the N- and C-terminal domains, resulting in different sized ATP-binding pockets [32].

### 2.3. Activation of the p38 Response

p38 kinases are activated by environmental and cellular stresses including pathogens, heat shock, growth factors, osmotic shock, ultraviolet irradiation, and cytokines. Moreover, various signaling events are able to stimulate p38 kinases, for example, insulin signaling. Interestingly, with respect to inflammatory responses, a number of studies have reported p38 regulation in macrophages treated with LPS, endothelial cells stimulated with TNF-*α*, U1 monocytic cells treated with IL-18, and human neutrophils activated with phorbol 12-myristate 13-acetate (PMA), LPS, TNF-*α*, and fMLP [33, 34]. It should also be noted that p38 activation in different cell types is dependent on the type of stimulus.

In addition, a number of studies have reported that distinct upstream kinases selectively activate p38 isoforms. p38 family kinases are all activated by MAP kinase kinases (MKKs). MKK6 activates all four p38 isoforms, while MKK3 can activate p38*α*, *β*, and *δ*, but not p38*γ* [35], and MKK4 activates p38*α* and *δ* [36]. This implies that p38 isoforms can be coactivated by the same upstream regulators and regulated specifically through different regulators.

### 2.4. p38 Deficiency

p38*α* deficiency affects placental development and erythropoietin expression and can result in embryonic lethality [37–40]. Tetraploid rescue of placental defects in p38*α* ^−/−^ embryos indicated that p38*α* was required for extraembryonic development, while it was not necessary for embryo development or adult mice survival. In accordance with the phenotype of p38*α* knockouts, knockout of two p38 activators, namely, MKK3 and MKK6, led to placental and vascular defects and induced embryonic lethality [41]. In contrast, p38*β* ^−/−^ mice were viable and exhibited no obvious health defects. Neither transcription of p38-dependent immediate-early genes, such as TNF-*α* and IL-1*β*, nor T cell development was influenced by the loss of p38*β* [42, 43]. Furthermore, mice harboring a T106M mutation in p38*α* resisted the drug inhibitory effect of collagen antibody-induced arthritis and LPS-induced TNF production, whereas the same mutation in p38*β* had the opposite effect [44], and p38*β* knockout mice responded normally to inflammatory stimuli. Single knockouts of either p38*γ* or p38*δ*, and even a double knockout, were viable [45]. However, reduced production of TNF-*α*, IL-1*β*, and IL-10 in stimulated macrophages isolated from p38 *γ*/*δ* null mice has been observed, which indicates that p38 *γ*/*δ* are important regulatory components of the innate immune response [46]. Taken together, these findings suggest that p38*α* is the critical isoform in inflammatory responses but that other subtypes also play important roles.

### 2.5. Regulation of p38-Activated Signaling

**Figure 1:**
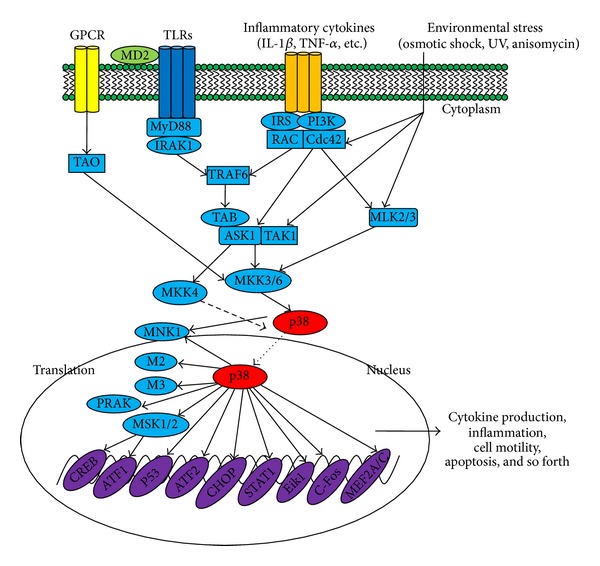
p38-regulated signaling pathways in inflammatory responses. Inflammation-derived cytokines such as TNF-*α* and IL-1, TLR ligands such as LPS, poly(I:C), and peptidoglycan, as a environmental stresses, stimulate the phosphorylation of p38, leading to the activation of transcription factors such as AP-1 family. Subsequent expression of inflammatory genes by these transcription factors mediates various inflammatory responses including cytokine production, migration, and apoptosis of macrophages, monocytes, and neutrophils.

Because p38 signaling can be activated by a variety of stimuli, the receptors and downstream pathways are diverse (Figure 1). MTK1, mixed lineage kinase (MLK) 2/3, apoptosis signal-regulating kinase (ASK) 1, and transforming growth factor *β*-activated kinase (TAK) 1 are all MKK kinases (MAP3Ks) that have been demonstrated to activate p38 signaling [47–54]. Furthermore, different kinases can mediate different signals. Among upstream proteins, Cdc42 and Rac are recognized as critical intermediates of p38 activity [55–57]. Many studies have also reported that p21-activated kinases (PAKs) can be stimulated by binding to Cdc42 and Rac* in vitro* and subsequently activate a p38 response [58–61]. In addition, Mst1, a mammalian homologue of Ste20, was reported to stimulate MKK6, p38, MKK7, and JNK [62]. However, there are no reports of the involvement of MTK1 and Mst1 in p38 responses in macrophages.

There are numbers of substrates downstream of p38 signaling pathways. MAP kinase-activated protein kinase 2 (M2) and M3 were the first p38 substrates identified [63, 64]. Phosphorylated M2 or M3 can activate a variety of substrates, such as small heat shock protein 27 (HSP27), CREB, and activating transcription factor (ATF) 1 [65, 66]. To date, several other proteins have also been identified as downstream substrates of p38, such as mitogen- and stress-activated kinase (MSK), p38-regulated/activated kinase (PRAK), and MAP kinase interaction protein kinase (MNK1) [67–70]. Various novel proteins have also been shown to be direct substrates of p38*α*, including Ahnak, Iws1, Grp78, Pgrmc, Prdx6, and Ranbp2 [71]. Additionally, TPL2/ERK1/2 has been shown to be downstream kinases controlled by p38 *γ* and *δ* isoforms [46].

Phosphatases that downregulate p38 activity have also been identified. Mitogen-activated protein kinase phosphatases (MKPs) can recognize MAPKs by recognizing the TXY amino acid motif and consequently dephosphorylate and deactivate them. MKP-1, MKP-4, MKP-5, and MKP7 can effectively dephosphorylate p38*α* and p38*β* [72–75]. However, right now, MKPs cannot dephosphorylate p38*γ* or p38*δ* as shown by other researchers [73, 76].

Several transcription factors in the nucleus can be phosphorylated and activated by p38 MAPKs, such as activating transcription factor 1 and 2 (ATF-1, ATF-2), myocyte enhancer factor 2 (MEF2), CCAAT/enhancer-binding proteins (C/EBPs), SRF accessory protein-1 (Sap1), p53, and E26 transformation-specific sequence-1 (ETS-1) [77–82] (Figure 1).

## 3. p38 Functions in Macrophage-Mediated Inflammatory Responses and Diseases

Macrophages are the first line of defense of organisms against pathogens. They represent a major cell population distributed in most tissues, and their numbers increase massively in inflammatory diseases. In particular, macrophages are critically involved in the pathogenesis of rheumatoid arthritis (RA) and produce a variety of proinflammatory cytokines and chemokines that contribute to cartilage and bone degradation. They are also the predominant cells in the synovial lining and sublining of patients with RA [83]. Macrophages also play a central role in the development of type 2 diabetic nephropathy. Macrophage accumulation in kidney, coronary arteries, nerves, and epiretinal membrane is regarded as one of major causing factors in terms of type 2 diabetic complications, including nephropathy, atherosclerosis, neuropathy, and retinopathy [84–88]. Components of the diabetic milieu, including high glucose, advanced glycation end products, and oxidized low-density lipoprotein, promote macrophage accumulation and activation within diabetic tissues [89]. Macrophage depletion studies have also demonstrated the crucial role of macrophages in the development of diabetic complications [89]. Moreover, macrophages play a pivotal role in the clearance of pulmonary pathogens. Alveolar macrophages (AM) constitute more than 90% of the cells present in bronchoalveolar lavage of naïve tissues [90]. AM can rapidly clear bacteria from airways and cellular debris, help to depress the immune characteristics of the airways, and aid in lung parenchyma modeling [90]. Furthermore, macrophages have significant roles in metabolic diseases, atherosclerosis, bowel disease, and liver fibrosis [91–94]. The fundamental roles of macrophages in inflammation highlight the need for macrophage-targeted studies and therapeutics.

Accumulating evidence suggests that p38 plays an essential role in macrophage-mediated inflammation. p38*α* is involved in the expression of proinflammatory mediators in macrophages such as IL-1*β*, TNF-*α*, PGE_2_, and IL-12 [95–97] as well as COX-2, IL-8, IL-6, IL-3, IL-2, and IL-1, all of which contain AU-rich elements (AREs) in their 3′ untranslated regions to which p38 binds [98]. Moreover, p38 can regulate the production of endothelial vascular cell adhesion molecule-1 (VCAM-1), which participates in cell proliferation and differentiation of the immune response [99]. Furthermore, p38 is associated with various inflammatory diseases, including endotoxin-induced shock, collagen-induced arthritis, granuloma, diabetes, and acute lung inflammation [100–103], as well as joint diseases, including synovial inflammation, cartilage damage, and bone loss [104]. In contrast, p38*β* and *δ* also play important roles in regulation of TPA-induced skin inflammation and tumor development [105, 106]. In addition, a large number of reports have suggested a close correlation between p38 and cell apoptosis, cell cycle progression, and differentiation [107–110].

## 4. Development of p38-Targeted Drugs as New Anti-Inflammatory Therapeutics

**Figure 2:**
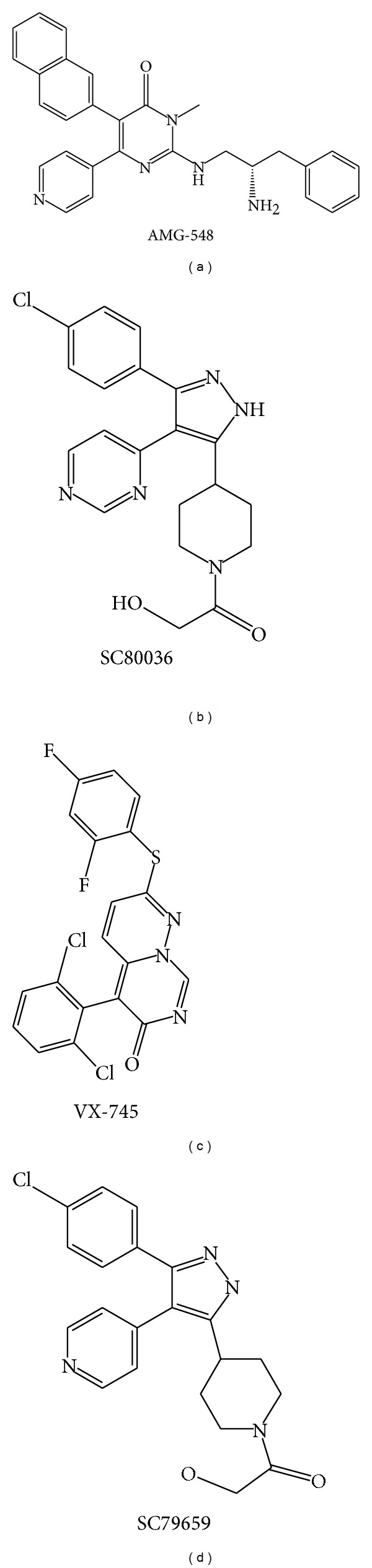
Chemical structures of representative novel p38 inhibitors. Promising therapeutic activities of these inhibitors against inflammatory diseases such as RA encourage the continuous progression for clinical trials.

p38 MAPK signaling plays a significant role in the inflammatory response and other physiological processes. A better understanding of the functional and biological significance of p38 in inflammation has led to the development of p38 inhibitors. Currently, a number of p38 inhibitors have been developed such as AMG-548, SC-80036, SC-79659, and VXs (Figure 2) [111]; however, few studies have reported their effects on macrophages.

### 4.1. Discovery

p38 signaling and specific p38 inhibitors were identified simultaneously. A series of pyridinyl imidazole anti-inflammatory agents, such as bicyclic pyridinyl imidazoles SKF-86002, SB203580, and SB202190 [15, 112–116], were first found to inhibit p38 activity [104, 117]. SB inhibitors can antagonize p38 by competing for the ATP-binding pocket, and it has been suggested that Thr-106 could be important for this interaction [115].

### 4.2. Crude Plant Extracts

Natural plant extracts that target p38 are promising therapeutics for the treatment of inflammatory diseases (Table 2). For example,* Scutellaria baicalensis* extract attenuates MAPK phosphorylation, especially p38 activity, resulting in inhibition of inflammatory mediators such as COX-2, iNOS, L-1*β*, IL-12, IL-6, IL-2, PGE_2_, and TNF-*α* in RAW 264.7 cells treated with LPS [118].* Phaseolus angularis* ethanol extract suppressed the release of PGE_2_ and NO in macrophages activated by LPS-, Poly(I:C)-, or pam3CSK through regulation of TAK1/p38 pathways and, moreover, it ameliorated gastritis induced by EtOH/HCl in mice, which implies a close relationship between p38 and gastritis [119].* Archidendron clypearia *extract suppressed the production of PGE_2_ in activated RAW264.7 and peritoneal macrophages, as well as gastritis lesions in mouse stomachs exposed to EtOH/HCl [28]. Unfortunately, p38 is not the only target of these extracts; they contain several other active ingredients and therefore are not good candidates for the development of p38-specific inhibitors. However, they are effective at treating inflammatory diseases because of their multiple targets and their ability to improve body's homeostatic defense responses [120–123]. Meanwhile, as reported previously [124], during covering years 1981–2006, of the 974 small molecule new chemical entities, 63% were naturally derived or semisynthetic derivatives of naturally occurring products, which indicate the importance of plant extract in the drug development [124]. In addition, we and other groups have found that various traditional plant extracts that target p38 kinase can reduce the symptoms of inflammatory diseases (unpublished data), such as gastritis, colitis, arthritis, and hepatitis [28, 29]. Plant extract data are summarized in Table 2.

### 4.3. Plant-Derived Compounds

Several compounds from natural products inhibit p38 activity and inflammatory responses (Table 3). Sugiol, an aditerpene that was isolated and purified from alcohol extracts of the bark of* Calocedrus formosana*, effectively decreased the production of intracellular reactive oxygen species (ROS), IL-1*β*, and TNF-*α* in LPS-stimulated macrophages through regulation of MAPKs [125]. Quercetin, a plant-derived flavonoid that is widely distributed in fruits and vegetables, strongly decreased the expression of the inflammatory cytokines iNOS and TNF-*α* by targeting both MAPK (ERK and p38) and I*κ*B*α* signaling pathways [126, 127]. Sulfur-containing compounds from garlic inhibited the production of NO, PGE_2_, and proinflammatory cytokines such as TNF-*α*, IL-1*β*, and IL-6 in macrophages by suppressing p38 transduction pathways [128]. A summary of natural products targeting p38 is provided in Table 3. These studies indicate that natural products inhibiting p38 activity exhibit strong anti-inflammatory properties, and are therefore potential therapeutic drug candidates for inflammatory diseases. Moreover, studies of natural compounds, in addition to elucidating why these extracts have strong anti-inflammatory effects, can also aid the design of novel p38 inhibitors to treat inflammatory diseases.

### 4.4. Novel Inhibitors

Pharmaceutical companies and researchers have worked hard to develop novel, safe, and specific p38 inhibitors. Based on the importance of p38*α* in inflammation, people have focused on inhibitors for this isoform rather than the other isoforms. ML3403, a SB203580 analogue, represses the expression of TNF-*α*, IL-6, and IL-8. It can bind to both active and inactive forms of p38*α* kinase, which may reduce asthma-induced airway inflammation and remodeling [129]. AS1940477 has been shown to inhibit the production of proinflammatory cytokines such as TNF-*α*, IL-1*β*, IL-6, PEG_2_, and MMP3 at very low concentrations. Moreover, it can reduce the enzyme activity of both p38 *α* and *β* but has no effect on 100 other kinases, including p38*γ* and *δ*. It has been shown in rats experiment that low doses of this compound can also reduce the expression of LPS- and Con A-stimulated proinflammatory cytokines, including TNF-*α* and IL-6 [130]. Pamapimod strongly suppresses p38 *α* and *β* activity and therefore the expression of TNF-*α*, IL-1*β*, and IL-6. It also shows high specific activity; when tested for binding to 350 kinases, it only bound to four other kinases in addition to p38. Furthermore, it can reduce clinical signs of inflammatory diseases, such as arthritis, bone loss, and renal diseases. Consistent with this, it inhibited TNF-*α* production in RA synovial explants and reduced bone loss in murine collagen-induced arthritis. Meanwhile, it increased pain tolerance in a rat model of hyperalgesia [131]. Examples of other newly synthesized compounds are GSK-681323 to treat rheumatoid arthritis, SCIO-469 to treat multiple myeloma and dental pain, and RWJ67657 that was developed as an anti-inflammatory drug, all of which inhibit p38 activity [98]. In summary, most of these inhibitors were designed based on the structure of SB203580 but show more specific and stronger activity. They are therefore promising therapeutic agents for inflammatory diseases.

### 4.5. Inhibitors in Human Clinical Trials

Based on the importance of p38 MAPK in disease development, inhibition of p38 was regarded as a promising therapeutic strategy to control inflammatory diseases. Right now, effectiveness of some p38 inhibitors is currently under evaluation in clinical trials to treat human diseases. For example, it has been reported that PH797804 and losmapimod were able to improve lung function parameters and to attenuate dyspnoea in patients with chronic obstructive pulmonary disease symptoms [132, 133]. Also, losmapimod was reported to reduce vascular inflammation in the most inflamed regions in patients with atherosclerosis [134]. Clinical and histological improvements linked to the inhibition of TNF-*α* level were clearly seen by p38 MAPK inhibitor adalimumab in lesional psoriatic skin [135]. Moreover, it was found that pamapimod can clearly alleviate various rheumatoid arthritis symptoms when coadministered with methotrexate [136]. Besides, there are still many other inhibitors which are ongoing clinical trials as summarized in Table 4 [137–140].

## 5. Summary and Perspectives

Inflammation has attracted great interest because of its significant role in several major diseases and the need to develop better ways to treat these diseases. Importantly, because inflammatory responses are largely mediated by macrophages, functional studies of macrophages in inflammation are crucial. Investigation of the roles of p38 MAPKs is particularly relevant as these are essential protein kinases in macrophage-mediated inflammatory responses. A number of studies have indicated that p38 plays a significant role in inflammatory diseases mediated by macrophages, and, as a consequence, several p38 inhibitors have been developed to treat inflammatory diseases. However, most of these inhibitors have shortcomings, such as low specificity, low efficacy, and high toxicity. As a result, new p38 inhibitors are urgently required. We are optimistic that novel and safe p38 inhibitors that possess strong anti-inflammatory properties will be developed in the near future to treat inflammatory diseases.

## Figures and Tables

**Table 1:** p38 family members and their functions in inflammatory responses.

p38 isoform (molecular weight, kDa)	Distribution in tissue	Expressing cells	Inflammatory responses	Reference
p38*α* (38)	Ubiquitous	Macrophages, neutrophils	Cytokine production (IL-1*β*, TNF-*α*, and IL-6); regulation of enzymes (iNOS, COX2); involvement of cell proliferation and differentiation; induction of cardiomyocyte apoptosis.	[21, 27, 73]

p38*β* (39)	Ubiquitous	Endothelial cells, T cells	Regulation of cell differentiation; induction of cardiomyocyte hypertrophy.	[21, 27, 73]

p38*γ* (43)	Skeletal muscle	Not detected	Muscle differentiation.	[25, 27, 73]

p38*δ* (40)	Lung, kidney, testis, pancreas, and small intestine	T cells, endothelial cells, and macrophages	Developmentally regulated; involvement of cell differentiation.	[26, 27, 73]

**Table 2:** Plant extracts that inhibit the p38 signaling in macrophages.

Plant	Action target of p38	Reference
*Archidendron clypearia *	Suppression of PGE_2_ production; amelioration of EtOH/HCl-induced gastritis	[28]
*Scutellaria baicalensis *	Inhibition of iNOS, COX-2, PGE_2_, IL-1*β*, IL-2, IL-6, IL-12, and TNF-*α* expression	[118]
*Phaseolus angularis *	Suppression of the release of PGE_2_ and NO; amelioration of EtOH/HCl-induced gastritis	[119]
*Artemisia vestita *	Inhibition of TNF-*α* release; beneficial for the treatment of endotoxin shock or sepsis	[141]
*Boswellia serrata *	Inhibition of TNF-*α*, IL-1*β*, and IL-6	[142]
*Hibiscus sabdariffa *	Suppression of nitrite, PGE_2_ release, and hepatic inflammation	[143]
*Clinopodium vulgare *	Suppression of NO production; MMP-9 activation	[144]
*Eriobotryae folium *	Suppression of LPS-induced NO and PGE_2_ production	[145]
*Elaeocarpus petiolatus *	Inhibition of the production of PGE_2_, TNF-*α*, and IL-1*β*	[146]
*Polygonum cuspidatum *	Inhibition of IL-6, TNF-*α*, NO, and PGE_2_	[147]
*Ginkgo biloba *	Inhibition of LPS-induced iNOS and COX-2 expression	[148]
*Lycium chinense *	Inhibition of LPS-induced NO, PGE_2_, TNF-*α*, and IL-6 production	[149]
*Hopea odorata *	Inhibition of NO, PGE_2_, and TNF-*α* release; amelioration of gastritis and ear edema	[150]

**Table 3:** Naturally occurring compounds that inhibit p38 signaling in macrophages.

Compound	Action target of p38	Reference
Sugiol	Inhibition of IL-1*β*, TNF-*α*, and ROS production	[151]
Quercetin	Inhibition of NO and TNF-*α*	[114]
Ajoenes	Inhibition of NO, PGE_2_, TNF-*α*, IL-1*β*, and IL-6 production	[116]
Ginsan	Enhanced phagocytic activity; downregulation of TNF-*α*, IL-1*β*, IL-6, IFN-*γ*, and IL-18	[152]
4-Methoxyhonokiol	Inhibition of iNOS and COX-2 expression; inhibition of dye leakage and paw swelling	[153]
Schisandrin	Suppression of NO production and PGE_2_ release	[154]
Rengyolone	Inhibition of iNOS and COX-2 expression	[155]
Pseudocoptisine	Inhibition of proinflammatory mediators such as iNOS, COX-2, TNF-*α*, and IL-6	[156]
Mycoepoxydiene	Inhibition of LPS-induced proinflammatory mediators including TNF-*α*, IL-1*β*, IL-6, and NO	[157]
Britanin	Suppression of NO, PGE_2_, TNF-*α*, IL-1*β*, and IL-6	[158]
Hyperin	Inhibition of NO production through suppression of iNOS expression	[159]
Carnosol	Inhibition of LPS-stimulated NO production; antioxidative activity	[160]

**Table 4:** p38 inhibitors under human clinical trials.

Compound	Clinical trials	Reference
PH797804	Chronic obstructive pulmonary disease (COPD)	[132]
Losmapimod (GW856553)	COPD; atherosclerosis	[133, 134]
Adalimumab	Antipsoriatic	[135]
Pamapimod	Rheumatoid arthritis	[136]
VX-745	Werner syndrome	[137]
BMS-582949	Rheumatoid arthritis	[138]
SB-681323	Percutaneous coronary intervention (PCI); neuropathic pain	[139, 140]

## Abbreviations

MAPKs: Mitogen-activated protein kinases
COX2: Cyclooxygenase-2
TNF-*α*: Tumor necrosis factor alpha
PGE_2_: Prostaglandin E_2_
NF-*κ*B: Nuclear factor kappa-light-chain-enhancer of activated B cells
AP-1: Activator protein-1
CREB: cAMP response element-binding protein
CSBP: Cytokinin-specific binding protein
PMA: Phorbol myristate acetate
LPS: Lipopolysaccharide
MKPs: MAP kinase phosphatases
MKKs: MAPK kinases
PAK: p21-activated kinases
HSP27: Heat shock protein 27
TLRs: Toll-like receptors
PRRs: Pattern recognition receptors
MNK1: MAP kinase interaction protein kinase
ATF-2: Activating transcription factor-2
MEF2C: Myocyte enhancer factor 2C
PRAK: p38-regulated/activated kinase
MSK: Mitogen- and stress-activated kinase
ARE: AU-rich elements
ICAM: Cell adhesion molecule.
